# Genetic Diversity of the KIR/HLA System and Outcome of Patients with Metastatic Colorectal Cancer Treated with Chemotherapy

**DOI:** 10.1371/journal.pone.0084940

**Published:** 2014-01-31

**Authors:** Valli De Re, Laura Caggiari, Mariangela De Zorzi, Renato Talamini, Vito Racanelli, Mario D’ Andrea, Angela Buonadonna, Vittorina Zagonel, Erika Cecchin, Federico Innocenti, Giuseppe Toffoli

**Affiliations:** 1 Translational Research, CRO National Cancer Institute, IRCCS, Aviano, Pordenone, Italy; 2 Epidemiology and Biostatistics, CRO National Cancer Institute, IRCCS, Aviano, Pordenone, Italy; 3 Internal Medicine and Clinical Oncology, University of Bari Medical School, Bari, Italy; 4 Medical Oncology Unit, San Filippo Neri Hospital, Rome, Italy; 5 Medical Oncology, CRO National Cancer Institute, IRCCS, Aviano, Pordenone, Italy; 6 Medical Oncology, Istituto Oncologico Veneto, IRCCS, Padova, Italy; 7 University of North Carolina, Institute for Pharmacogenomics and Individualized Therapy, Eshelman School of Pharmacy, School of Medicine, Lineberger Comprehensive Cancer Center, Chapel Hill, North Carolina, United States of America; Ohio State University Medical Center, United States of America

## Abstract

**Objective:**

To explore genes of the killer-cell immunoglobulin-like receptor (KIR) and of the HLA ligand and their relationship with the outcome of metastatic colorectal cancer (mCRC) patients treated with first-line 5-fluorouracil, leucovorin, and irinotecan (FOLFIRI).

**Methods:**

A total of 224 mCRC patients were screened for KIR/HLA typing. The determination of the KIR/HLA combinations was based upon the gene content and variants. Genetic associations with complete response (CR), time to progression (TTP) and overall survival (OS) were evaluated by calculating odds and hazard ratios. Multivariate modeling with prognostic covariates was also performed.

**Results:**

For CR, the presence of KIR2DL5A, 2DS5, 2DS1, 3DS1, and KIR3DS1/HLA-Bw4-I80 was associated with increased CR rates, with median ORs ranging from 2.1 to 4.3, while the absence of KIR2DS4 and 3DL1 was associated with increased CR rates (OR 3.1). After univariate analysis, patients that underwent resective surgery of tumor, absence of KIR2DS5, and presence of KIR3DL1/HLA-Bw4-I80 showed a significant better OS (HR 1.5 to 2.8). Multivariate analysis identified as parameters independently related to OS the type of treatment (surgery; HR 2.0) and KIR3DL1/HLA-Bw4-I80 genotype (HR for T-I80 2.7 and for no functional KIR/HLA interaction 1.8). For TTP, no association with KIR/HLA genes was observed.

**Conclusion:**

This study, for the first time, evidences that the genotyping for KIR-HLA pairs are found predictive markers associated with complete response and improves overall survival prediction of FOLFIRI treatment response in metastatic colorectal cancer. These results suggest a role of the KIR/HLA system in patient outcome, and guide new research on the immunogenetics of mCRC through mechanistic studies and clinical validation.

## Introduction

The innate immune system is the first line of defense in response to tumor cell. Natural Killer (NK) cells perform an important role in this response with their ability to kill tumor cells, produce cytokines, and cross-talk with the adaptive system. Interactions between killer-cell immunoglobulin-like receptors (KIR)-receptors and human leucocyte antigen (HLA)-ligand regulate the response of NK cells, resulting in a multitude of KIR/HLA combinations and different NK effects. Modification of tumor-cell due to chemotherapeutic-treatment may also contribute to a difference in NK-response.

Similar to other combination regimens in metastatic colorectal cancer (mCRC), treatments based upon FOLFIRI (5-fluorouracil, leucovorin and irinotecan), used in about 60% of first-line treatment in Europe [1], remain ineffective in a significant proportion of patients [2]. Immunotherapy aimed at enhancing natural antitumor T-cell immunity in patients affected by advanced malignancies are currently being implemented in the clinic with promising results, often showing a synergic effect with chemotherapy [3]. In order to optimize therapeutic protocols and monitor the effectiveness of such therapies, acknowledgement of the exact mechanisms that modulate action of the host immunity resulting in an effect on patient outcome are not entirely clear and could involve different T-cell receptors and immune cells. Further work is needed to fill the remaining gaps in our knowledge.

Pathological staging is the only prognostic classification used in clinical practice to select patients for chemotherapy. Gene expression profiling, which however require primary tumor samples, might contribute to the classification and prognostic value prediction of colon cancer [4]. Additional factors, preferably easily available, to identify patients at the highest risk for recurrence (prognostic factors) as well as to predict those most likely to benefit from chemotherapy (predictive factors) are usefull to improve the selection of patients for adjuvant chemotherapy. Moreover novel Response Evaluation Criteria in Solid Tumor (WHO criteria), designed immune-related response criteria (irRC) to evaluate antitumor responses to chemotherapeutic agents, is increasingly resulting in a better assessment of anti-tumor agents [5] for more frequent use of immunotherapy and immune-chemotherapy in cancer patients [6]; [7].

Different types of infiltrating immune cells have different effects on tumor progression [8]. NK cells can affect tumor progression and could represent a surrogate marker of response of the host immunity against cancer cells, including CRC [9]; [10]. NK cell infiltration in situ was found to be minimal within surgically resected CRC samples, as opposed to the higher numbers of NK cells in the adjacent normal mucosa [11]. Accumulating evidence indicates that overexpression of the HLA-E on CRC cells leads to inhibition of NK cell-mediated anti-tumor response by engaging CD8 T cells and NK cell subsets [12].

NK cells, traditionally considered as part of the innate immune system, have also been shown to share many functions with adaptive immunity [13]; [14]. Contribution of KIR/HLA interaction to signaling in NK cells are not fully understood [15], but their importance has been underscored by several recent genetic studies which have linked KIR/HLA-combinations with the outcome of various diseases, and with some clinical response to treatment, including the one in this study. NK cells preferentially kill target cells that express few or no HLA class-I molecules on their surface. The activity of NK cells is regulated by a balance of transducer signals through activating and inhibitory receptors [16].

Most NK receptors appear to belong to a family known as KIRs. They are expressed in NK cells and in a small subset of T lymphocytes. The KIR receptors are either inhibitory (with long (L) tails, i.e. KIR2DL1) or activating (with short (S) tails, i.e. KIR2DS1). A distinct KIR typically interacts with a specific allotype of an HLA class-I molecule [15]; [17]. Each NK cell can express several different inhibitory and/or activating receptors that function independently from each other. Consequently, there is a relatively high level of pleiotropy and heterogeneity of KIRs in humans. The independent expression of polymorphic KIR genes and highly polymorphic HLA ligands determines the final NK cell function [15]; [18].

Cytotoxic agents can activate different pathways of cell death, inducing distinct peptide patterns of HLA cross-presentation of tumor-released antigens to cytotoxic T-cells, including NK cells [19]. A higher rate of tumor-associated antigen uptake by HLA-mediated antigen-presenting cells has been reported after the administration of 5-FU [20]. The anti-tumor effects of chemotherapy could be further enhanced by NK cell functions [21]–[25].

Because of the emerging role of the immune system in solid tumor biology [26], the potential role of immune effectors in mediating the efficacy of therapy [3]; [25] and the lack of clinical information on the role of the KIR/HLA system in mCRC, we aimed to: 1) characterize the genetic profile of the KIR/HLA system in an homogeneous group of mCRC patients treated with FOLFIRI, and 2) determine the effect of the patient genetic makeup of the KIR/HLA system on response rate and survival. For this purpose, we developed a KIR/HLA pair genotyping to characterize the spectrum of NK-related immune genes in CRC response to FOLFIRI treatment in univariate and multivariate analysis. We discuss here the studies relating to highly polymorphic KIR/HLA interactions with an emphasis on how these genes may regulate NK-function towards tumor response and survival of treated-patients.

## Methods

### Patients and Treatment

We used outcome data collected from a previous study, and eligibility criteria and FOLFIRI treatments have been previously described in details [27] For this study we selected patients with a stage IV and recurrent CRC. Patients were treated in first line with FOLFIRI only, and no biological therapies were allowed. The patients evaluable for objective response, time to tumor progression (TTP) and overall survival (OS) were 224.

The median duration of follow-up was 21.73 months (range: 1.07–91.8). Response rates were as follows: complete response (CR, n = 15), partial response (PR, n = 80), stable disease (SD, n = 62), and progressive disease (PD, n = 67). Tumor response was assessed using the WHO criteria as previously reported [28]. OS was defined as the time from the first drug administration to date of death or last follow-up. Patients alive at the last follow-up were censored. TTP was defined as the time from the date of the initial therapy to the earliest date of tumor progression or last follow-up. The median OS was 21.7 months (range: 1.07–91.8). The median TTP was 8.5 months (range: 0.7–41.6). Patient characteristics are shown in Table 1.

**Table 1:** Characteristics of the mCRC patients treated with FOLFIRI.

Characteristics	N. 224	(%)
**Sex**		
Male	153	(64.7)
Female	79	(35.3)
**Age (years)**		
<59	80	(35.7)
59–65	69	(30.8)
>65	75	(33.5)
**Tumor location**		
Rectum	67	(29.9)
Right-colon	88	(39.3)
Left-colon	69	(30.8)
**TNM stage at diagnosis**		
I–II	22	(9.8)
III	54	(24.1)
IV	148	(66.1)
**Radical surgery$**		
Yes	184	(82.1)
No	40	(17.9)
**Tumour response**		
CR	15	(6.7)
PR	80	(35.7)
SD	62	(27.7)
PD	67	(29.9)
**Adjuvant therapy**		
Yes	67	(29.9)
No	157	(70.1)

**$ Radical surgery:** Surgical resection of locally recurrent cancer.

### KIR and HLA Genotyping

DNA was extracted from blood using the EZ1 Qiagen Italy kit as previously described [29]. Patients were genotyped for the presence of the KIR genes (2DL1-4, 2DL5A and 2DL5B, 3DL1-3, 2DP1, 2DS1-3, 2DS4, 2DS5, 3DP1, and 3DS1) using a multiplex polymerase chain reaction-sequence specific primer reaction, optimized in our laboratory [30].

High resolution HLA class-I analysis was performed by PCR sequence-based typing with primers specific for loci A, B and C and the use of the Assign SBT software (version 3.27b) [30]. KIR and HLA gene frequencies were representative of those of a Caucasian population [31] and were comparable to those found in our series of non cancer blood donors (data not shown).

### Analysis of the KIR-HLA Genes

The KIR gene content shows a high degree of variation, in terms of number and type of genes present. Two main KIR haplotype groups have been described, based on gene content, and are designated as A and B [32]; [33]. The group A haplotypes have a simple and constant gene content, represented by genes encoding inhibitory receptors KIRs 2DL1, 2DL3, 2DL4, 2DS4, 3DL1, 3DL2 and 3DL3. In contrast, the group B haplotypes have more variable and greater gene content, involving genes encoding distinctive inhibitory receptors and a variety of activating receptors.

The associations of KIRs with their cognate HLA ligands were established on the basis of predicted KIR/HLA combinations, according to a standard classification [34]. HLA-A, -B and -C molecules were placed into 3 groups (HLA-C1, -C2, and Bw4) based on the amino acid sequences determining the KIR-binding epitope. HLA-C allotypes with asparagine at position 80 (HLA-C1) are ligands for the KIRs 2DL2, 2DL3, and 2DS2; HLA-C allotypes with lysine at position 80 (HLA-C2) are ligands for the KIRs 2DL1 and 2DS1. In the third group, HLA-A and -B allotypes with the Bw4 epitope are ligands for KIRs 3DL1 and 3DS1 and are distinguished by substitutions at position 77, 80, 81, 82 and 83 in the C terminus of the HLA class I α1 domain; HLA-A*03 and *11 are ligands for KIR3DL2 and HLA-C*04 is a ligand for KIR2DS4 [35].

### Statistical Analysis

Odds ratios (OR) and 95% confidence intervals (CI) were used to investigate the association between KIR/HLA genes and tumour response (CR, PR, SD and PD). TTP and OS were computed by using the Kaplan-Meier method, and the log-rank test was used to test the differences between subgroups. Cox proportional hazard regression models were used to calculate hazard ratios (HR) and 95% CIs. Fisher’s exact test and chi-squared analysis for trend were also used to test the effect of KIRs and HLAs on CR. The independence of the genetic associations from tumor and clinical characteristics was tested by multivariate modeling of covariates, including sex, age, tumor location (rectum; right colon; left-colon), TNM stage at diagnosis, radical surgery (surgical resection of locally recurrent cancer). Results were considered to be statistically significant when p<0.05 (two-sided). All analyses were carried out by using SAS 9.2 statistical software (SAS Institute, Inc., Cary, NC).

### Ethics Committee Approval

The study was coordinated and sponsored by the Centro di Riferimento Oncologico, National Cancer Institute, Aviano, Italy, accordantly to Declaration of Helsinki Principle. The use of blood sample collection was approved by CRO, institutional Board: RC linea 4. All patients provided written informed consent for genetic analysis before entering the study, this consent was considered sufficient and approved by the Institutional Review Board.

## Results

### Associations of HLA/KIR with Complete Response Rates

**Figure 1:**
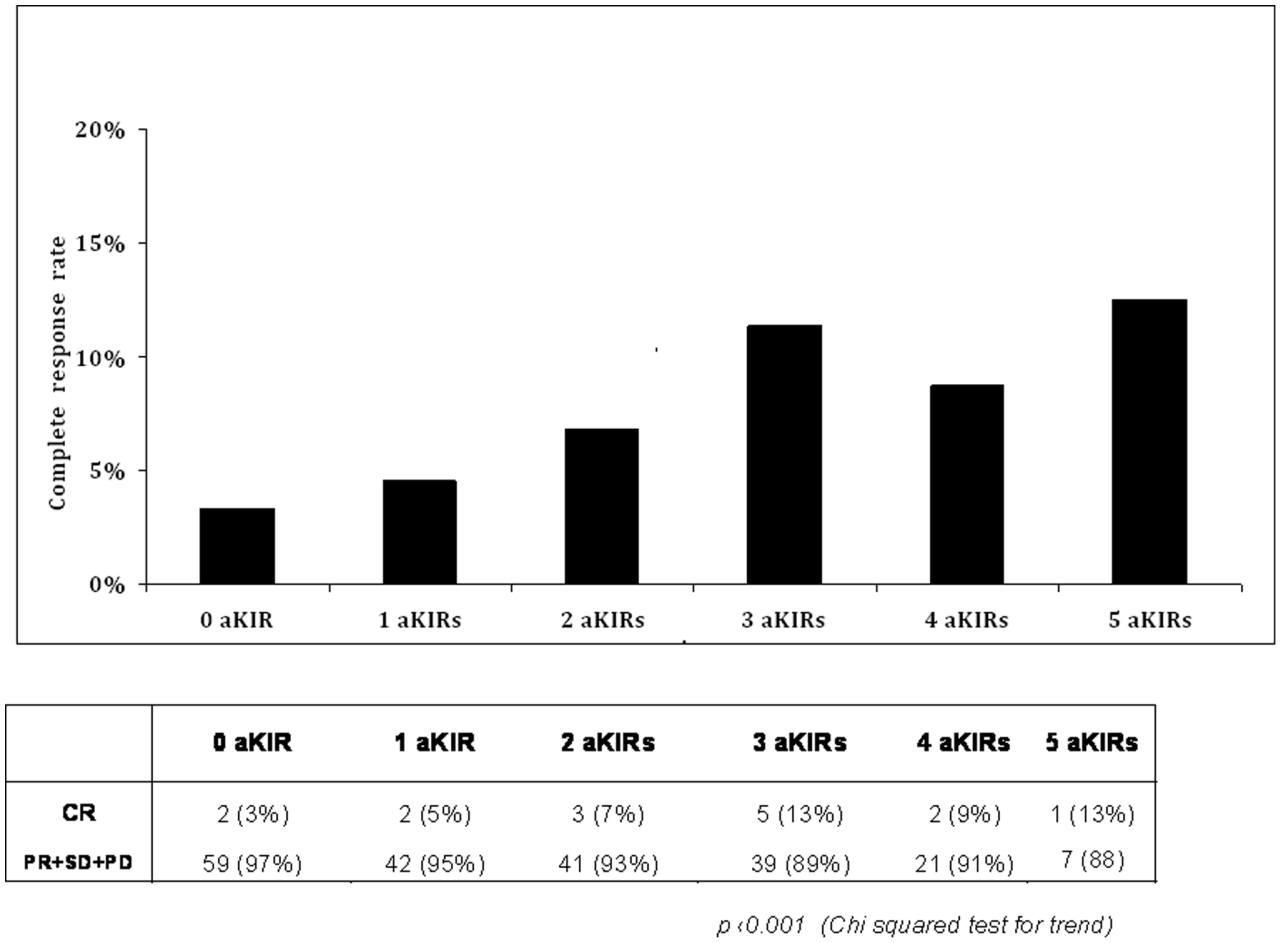
Association between the number of activating KIR (aKIR) genes and complete response (CR) rate. The KIR gene analysis shows a trend (chi squared test for trend, p<0.001) between an increasing number of aKIR genes (KIR2DS3 and KIR2DS5; ligand unknown) or aKIR/HLA-ligand (KIR2DS1/HLA-C2, KIR2DS2/HLA-C1, KIR2DS4full/HLA-Cw*04, KIR3DS1/HLA-Bw4) and the rate of CR.

Presence of KIR2DL5A, 2DS5, 2DS1, 3DS1 and KIR3DS1/HLA-Bw4-I80 was associated with improved CR rates (CR versus PR+SD+PD) with ORs ranging from 2.1 to 4.3 (p<0.05, Table 2). Conversely, absence of KIR2DS4 and 3DL1 was associated with an improved CR (OR 3.1, 95% CI 0.0–0.5, p 0.001, Table 2). KIR haplotype analysis indicated that the B/B haplotype, including KIR2DL5A, 2DS1, 2DS5, and 3DS1, but not KIR2DS4, was more frequent in patients with CR (33.3%) than in other patients (4.8%) (OR 5.45, 1.5–19.1, p 0.013). A/A and A/B haplotypes contain only KIR2DS4 as the single activating KIR gene, in agreement with the concept that activating KIR (aKIR) genes are usually associated only with the group B haplotype, an increasing number of aKIR genes (with their cognate HLA carrier) was associated with improved CR (p<0.001, Fig. 1). None of the patients and tumor characteristics listed in Table 1 was associated with CR rates (p>0.05, data not shown).

**Table 2:** Association between KIR/HLA and complete response (CR) rate.

	CR%	PR+SD+PD%	OR	(95% CI)	p
**KIR2DL5A**					
–	26.7	59.8	1		
+	73.3	40.2	4.1	(1.3–13.3)	0.02
**KIR2DS5**					
–	40.0	67.0			
+	60.0	33.0	3.0	(1.0–8.9)	0.04
**KIR2DS1**					
–	26.7	59.8	1		
+	73.3	40.2	2.1	(1.3–13.3)	0.02
**KIR3DS1**					
–	26.7	60.8	1		
+	73.3	39.2	4.3	(1.3–13.8)	0.02
**KIR2DS4**					
+	33.3	6.2	1		
–	66.7	93.8	3.1	(0.0–0.5)	0.001
**KIR3DL1**					
+	66.7	93.8	1		
–	33.3	6.2	3.1	(0.0–0.5)	0.001
**KIR3DS1/HLA-Bw4**					
−/−	46.7	74.2	1		
+/T80	13.3	9.6	2.2	(0.4–11.4)	0.34
+/I80	40.0	16.3	3.9	(1.2–12.4)	0.02

CR: complete response; PR: partial response; SD: stable disease; PD: progressive disease. + and − refer to presence and absence of a gene (respectively).

**Figure 2:**
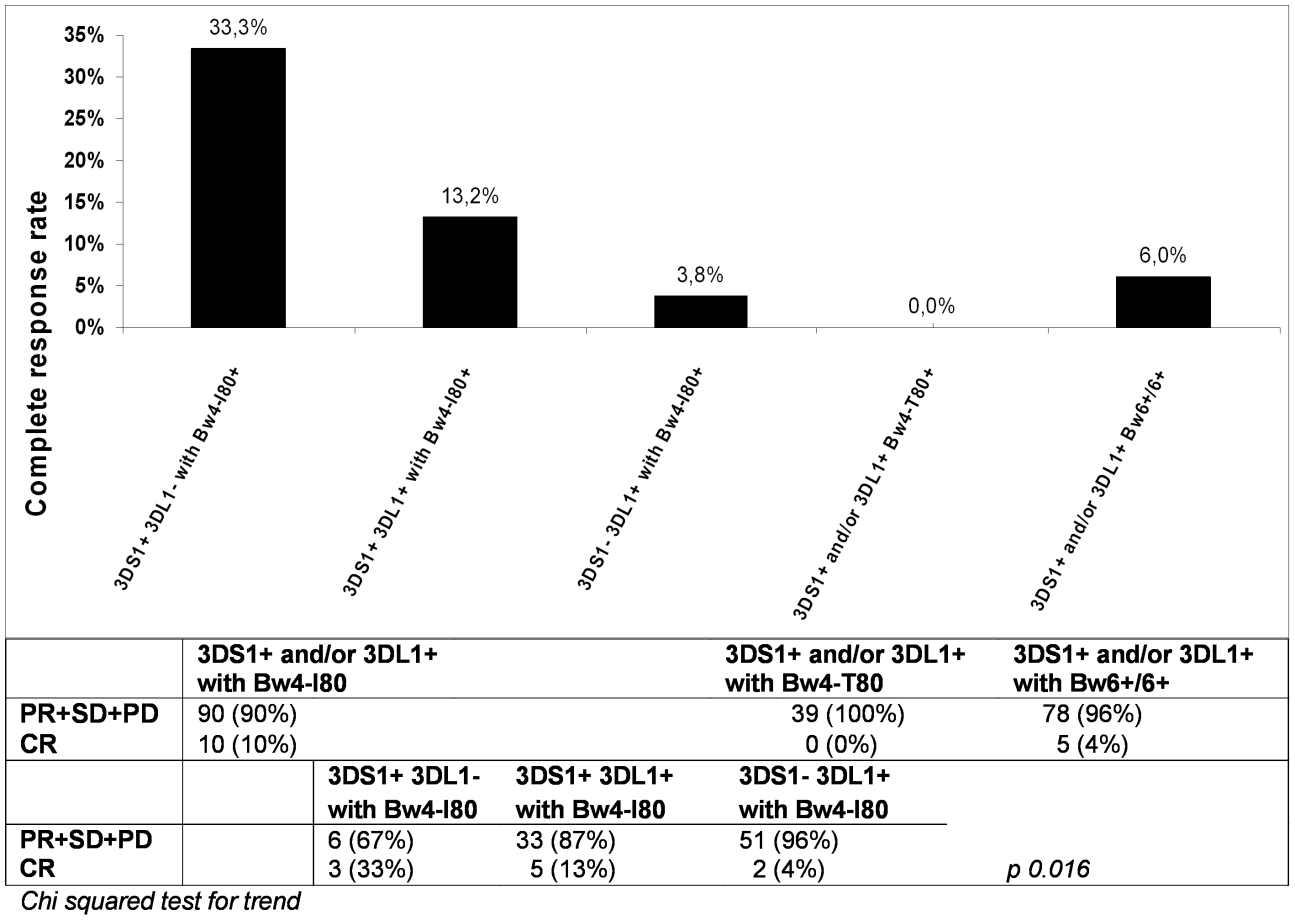
KIR3D and HLA-B ligands and their association with the CR rate. The NK effector functions are determined by the cumulative signal generated via the ligation of activating and inhibitory NK cell receptors with their putative HLA ligand on target cells. Activating (KIR3DS1) and inhibitory (KIR3DL1) receptors are genes of a same locus and have similar HLA-B ligand specificity with thus a possible balancing role in the NK response. The KIR3D receptor can recognize the HLA-B molecules carrying the Bw4 common epitope (Bw4-I80 and Bw4-T80 variants), but not the Bw6 motif. The figure shows a greater frequency of patients with CR in patients carrying KIR3Ds with the Bw4-I80 ligand (10%) than in patients with Bw4-T80 (0%) or Bw6/6 (4%). Among the HLA-Bw4-I80 ligand, the presence of the KIR3DS1 gene is associated with a trend towards improved CR rate (Chi square test for trend, p 0.016). + and – indicate presence or absence of the gene, respectively.

Analysis of KIR and their HLA ligands genotyping, showed a higher CR rate when combining KIR3DS1 with HLA-Bw4-I80 than when combined either with HLA-Bw4-T80 or HLA-Bw6 (not recognized by KIR3Ds) or in case of absence of the KIR3DS1 gene, respect to other patients (PR+SD+PD) (Table 2). Among different KIR genes, the activating KIR3DS1 and the inhibitory KIR3DL1 segregate as alleles of the same KIR3DL1/S1 locus and KIR3DL1 and putatively KIR3DS1 are receptors for a same subset of HLA-B alleles. Within the HLA-Bw4-I80 ligand, the 3 different combinations of KIR3DS1 and KIR3DL1 (i.e. presence/absence, presence/presence, absence/presence, respectively) were suggestive of a trend for increased CR rate in the presence of KIR3DS1 (p 0.016, Fig. 2).

### Associations of HLA/KIR with Overall Survival and Time to Progression

**Figure 3:**
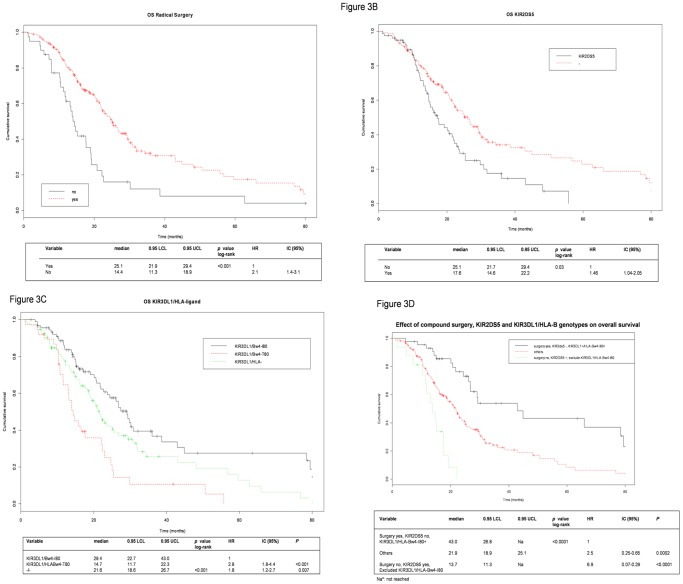
Kaplan-Meier survival curves (truncated at 80months) A) Kaplan-Meier plots of OS stratified by radical surgery Yes and No refer to presence or absence of radical surgery, respectively. **B) Kaplan-Meier plots of the association between KIR2DS5 and OS.** OS curves of patients with KIR2DS5 (solid black line), or without the gene (−, red dashed line). The implication of this result could not be further investigated as the ligand of KIR2DS5 has not been identified yet. **C) Kaplan-Meier plots of the association between KIR3DL1/HLA-B and OS.** OS curves of patients with KIR3DL1/HLA-Bw4-I80 (solid black line), KIR3DL1/HLA-Bw4-T80 (dashed red line), or without either one (−/−, green dashed line). **D) Kaplan-Meier plots of the association between a combination of surgery, KIR2DS5 and KIR3DL1/HLA and OS.** Yes and No refer to presence or absence of radical surgery, respectively. − refers to the absence of the gene. Kaplan-Meier method, and log-rank were used to test the differences between subgroups. 0.95 LCL and 0.95 UCL; lower and upper 95th percentile, respectively; CI (95%); 95% confidence interval.

Among patient characteristics, only surgical resection of locally recurrent cancer improved the OS rates in comparison with patients who did not have surgical resection (median 25.1 vs 14.4 months, HR 2.1, p<0.001, Figure 3A, Table 3 and Table S1).

**Table 3:** Association between KIR/HLA and overall survival (OS).

deaths/total %		Median OS Months (95% CI)	HR (95%CI)	*p*	HR (95%CI)	*p*	HR (95%CI)	*p*
**Radical surgery**								
Yes	59.2	25.1 (21.9–29.4)	1†		1†		1†	
No	82.5	14.4 (11.3–18.9)	2.1 (1.4–3.1)	*<0.001*	2.0 (1.3–2.9)	*<0.001*	2.0 (1.3–3.0)	*<0.001*
**KIR2DS5**								
−	60.3	25.1 (21.7–29.4)	1†		1†			
+	69.2	17.6 (14.6–22.2)	1.5 (1.0–2.1)	*0.03*	1.3 (1.0–1.9)	*0.10*		
**KIR3DL1/HLA-Bw4**								
+/I80	52.7	29.4 (22.7–43.0)	1†				1†	
+/T80	73.2	14.7 (11.7–22.3)	2.8 (1.8–4.4)	*<0.001*			2.7 (1.7–4.2)	*<0.001*
−/−	65.6	21.6 (18.6–26.7	1.8 (1.2–2.7)	*0.007*			1.8 (1.2–2.7)	*0.006*

+ and − refer to presence and absence of a gene (respectively).

† Reference category.

Among genotypes (Table S1), the presence of KIR2DS5 gene (Figure 3B) and KIR3DL1/HLA-Bw4-I80 (Figure 3C) was associated with improved OS, while KIR3DL1/HLA-Bw4-T80 (HR 2.8, p<0.001) or a non interacting KIR3DL1 receptor (−/−) (HR 1.8, p 0.007) was associated with worse OS. Median OS was 25.1 months in KIR2DS5 patients, 29.4 months (22.7–43.0) in KIR3DL1/HLA-Bw4-I80 patients, 14.7 months (11.7–22.3) in KIR3DL1/HLA-Bw4-T80 patients, and 21.6 months (18.6–26.7) in patients without KIR3DL1/HLA-Bw4 (either I80 or T80 or absence of KIR3DL1).

In multivariate analysis including the only clinical characteristic associated with OS (radical surgery) and KIR/HLA genotypes, surgery and KIR3DL1/HLA-Bw4-I80 retained an independent effect on OS (HR 2.0, p<0.001 and HR 2.7–1.8, p<0.001–0.006, respectively) (Table 3). The effect of compound patients who had (or not) radical surgery, who had (or not) KIR2DS5 gene that is partially in linkage disequilibrium with KIR3DL1 (http://www.allelefrequencies.net/kir6010a.asp. Accessed 2012 ctober 17), and KIR3DL1/HLA-B pairs on the OS is illustrated in Figure 3D. Median OS was 43.0 months in patients treated with surgery, without KIR2DS5 but KIR3DL1/Bw4-I80+ genotype and 13.7 months in patients without surgery treatment and having a genotype including KIR2DS5 but no KIR3DL1/HLA-Bw4-I80 pair genes.

For the analysis of TTP, neither individual KIR genes nor combinations of KIR/HLA genes were significantly associated with TTP (p>0.05, data not shown).

## Discussion

The study, for the first time, has investigated the clinical relevance of KIR/HLA gene combinations in mCRC patients and FOLFIRI treatment. Depending upon the KIR/HLA combination, tumor response and OS differed among patients who had similar clinical and tumor characteristics. Only two studies have investigated the distribution of KIR and HLA genes in CRC specimens [36]; [37], but these studies analyzed only KIR/HLA frequencies in cancer patients compared to normal control subjects with the aim to test the impact of KIR/HLA status on individual’s susceptibility to CRC.

Advances have been made in the last years in metastatic colorectal cancer treatments, with the introduction of novel molecularly targeted agents like antibodies against the VEGF or the EGFR targets; nonetheless rate of long survivors are low. NK cells provide first-line defense against malignantly transformed cells [38]; [39] and recent evidence of the use of antibodies blocking the immunosuppressive receptor programmed cell death 1 (PD-1) pathway underscored the central role of reinduction of host immune system and of potentiating the NK-cell mediated cytotoxicity in several tumor prognosis [40]; [41] including CRC [3]. Moreover, some of the clinically approved therapeutic antibodies to treat cancer, such as cetuximab and rituximab, are considered to function partially through triggering NK cell mediated ADCC activity [42]. Overall, despite the central role of NK-cells in host immune responses and the fact that the KIR/HLA gene system is the principal receptor system able to modulate NK cell function, KIR/HLA genotypes on the clinical outcome in mCRC has been essentially unexplored.

The analysis of the KIR/HLA genetic make-up of mCRC patients proposes that presence of KIRs 2DL5A, 2DS5, 2DS1 and 3DS1/HLA-Bw4 ligand pair and absence of the KIR2DS4 and 3DL1 (Table 2, Figs 1–2) might influence the likelihood of achieving a radiologic response after chemotherapy. Thus, in our series patients showing a more activator haplotype (eg haplotype B/B), and especially KIR3DS1/HLA-Bw4-positive NK cells in absence of their counteracting inhibitor KIR3DL1 receptor gene, should be more prone to cytolytic activity and CR than other patients.

State of the art for KIR/HLA genotypes indicate that KIR/HLA combinations inhibit NK cells with different intensities [43]; [44] and that NK-cells will become activated when inhibition is removed, so activation must involve stimulatory receptors [45]. Furthermore, a decrease trend for NK cytolitic activity was found against recipient target cells, which have HLA-C2 (strong), HLA-C1 (modest), HLA-Bw4 (weak cytolitic activity), and then having no missing-self ligand [46]; [47]. Therefore, NK cells having inhibitory KIR receptors with a lower avidity to HLA-ligand (thus, having a decreased inhibitory function respect to other KIRs) and having many activator KIR receptors may show an increased NK-mediated cytolysis of target cells. For example, the presence of the strong activating receptor KIR2DS1 with its cognate HLA-C2 ligand were more prone to kill HLA-C2+ cells [48] and this was associated with increased progression-free survival (PFS) in chronic lymphocytic leukemia patients [49]. In addition, the block of inhibitory KIR/HLA interaction has been showed to decrease leukemic progression in an *in vivo* animal model [50]. In patients treated with autologous hematopoietic stem cell transplantation for high risk for neuroblastoma, the absence of functional inhibitory KIR/HLA combination was associated with a lower risk of death and progression [51]. An increased NK-cell mediated cytotoxic activity obtained by blocking the inhibitory receptor PD-1 upexpressed on NK-cells of patients with several solid tumors including CRC [52] was also associated with a durable cancer regression [3]. Our results are thus consistent with the hypothesis that the presence of activator KIRs (i.e. high number of aKIRs and the presence of KIR3DS1/HLA-Bw4-I80, Fig. 1 and 2) has a beneficial effect on tumor response. It is known that chemotherapy can induce distinct patterns of HLA cross-presentation of tumor-released antigens to cytotoxic T-cells [20]; [53], and that the anti-tumor effects of chemotherapy could be modified by NK cells [21]; [22]; [25]. Down-regulation of the antigen presentation by the HLA machinery and a reduction in the number of T-cells infiltrating the tumor site have been associated with worse prognosis [54]; [55]. Moreover, NK cell counts in tumor beds [11]; [12]; [53]; [56] have been shown to be favorable prognosticators, also in CRC. Our data seem to complement these observations, suggesting that, depending on a more responsive NK cell phenotype (number of aKIRs) and specific activatory/inhibitory KIR/HLA pairs (e.g. KIR3D/HLA-Bw4), tumor response could be more or less effective, leading to a different degree of reduction in the size of primary tumors. At the cellular level, these effects could be mediated by an increased accumulation of activator KIR receptors on NK cells and effective HLA-ligands in tumor cells enhancing NK-mediated tumor immunity. By analogy to the findings, in other situations, the result of cytolysis may be more effective when a low dose of target cells is present, but not sufficiently effective in the case with high-dose of target cells in whom the innate immune response is likely overwhelmed [57]. By the same way it is plausible that some peptides (new or only more abundant) may be presented by HLA to KIR in the course of cancer growth, or even edited due to mCRC treatment, and thus that peptides/HLA complexes may influence the NK cell function at site of the tumor [58]; [59]–[64].

**Figure 4:**
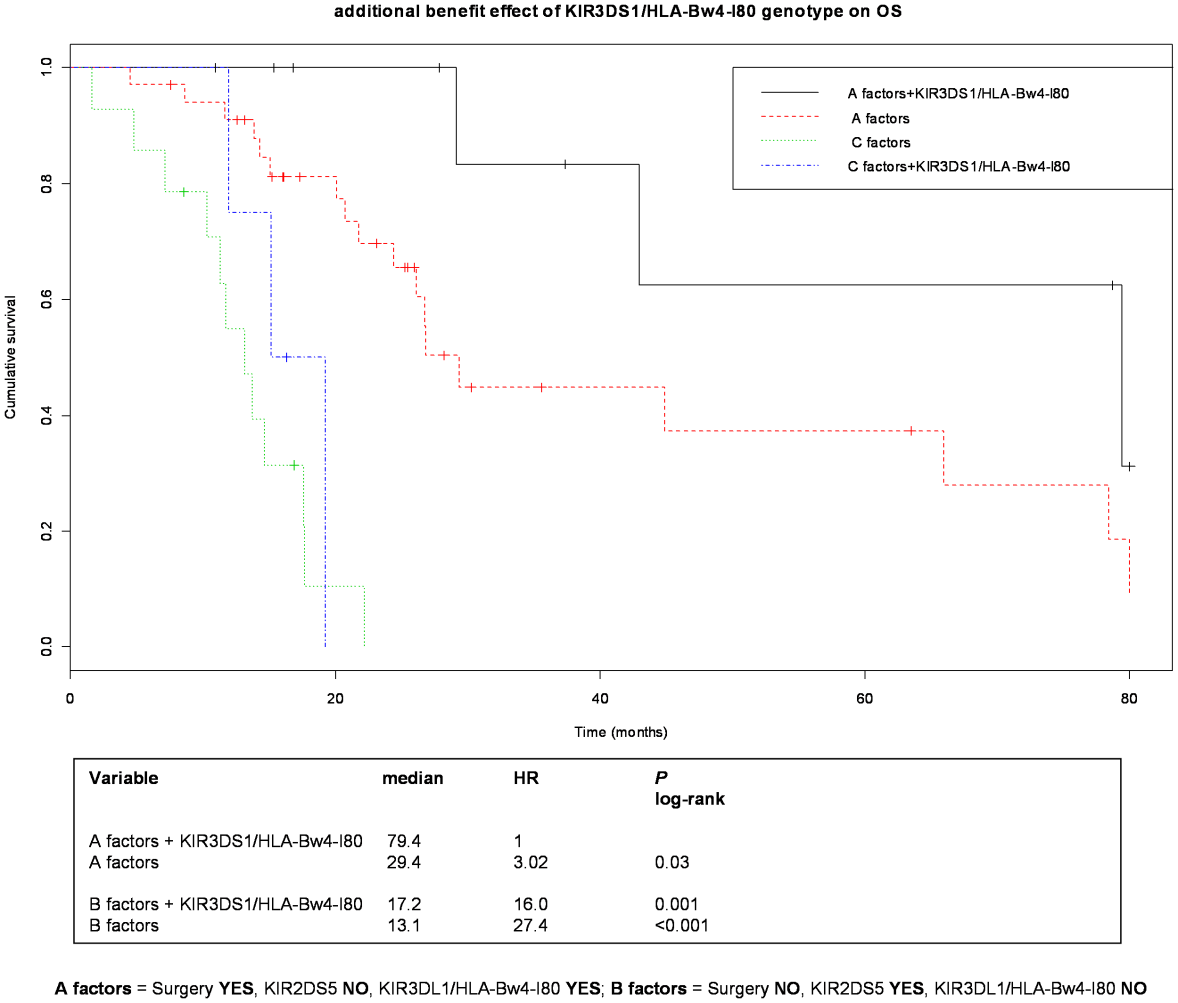
Presence of KIR3DS1/HLA-Bw4-I80 genotype associated with CR, improves the survival of patients stratified on combination of genes and surgical treatment. OS Kaplan-Meier survival curves (truncated at 80months) of patients stratified on patients with the identified better (A factors) and worse (B factors) prognostic combination of genes and surgical treatment.

Among characteristics of patients, only surgery ups OS. Data from KIR and KIR/HLA genotype by univariate analysis identified two KIRs associated with survival previously found associated with CR; the KIR2DS5 and KIR3DL1. After multivariate comparison with surgery, only the KIR3DL1/HLA-Bw4-I80 remain statistically significant, indicating that KIR3DL1/HLA-Bw4-I80 genotype was strongly predictive of survival independently of surgery (Table 3). Since i) to nowadays the ligand of KIR2DS5 is unknown, ii). KIR2DS5 is in positive linkage disequilibrium with the KIR3DL1 gene (LD 30), iii) KIR3DL1 and 3DS1 are genes of a same locus, iv) KIR3D genotype and surgery are independent factors (Table 3), we evaluated their effects by composite KIR genes in OS Kaplan Meier plots of patients previously treated with combined surgery and chemotherapy or treated only with chemotherapy because surgery was not indicated. Results showed patients with a better OS from the other ones (median OS 79.4 to 22.9 months); the best increased OS was found when both KIR3DL1 and 3DS1 genes of the same locus were in the presence of their HLA counterpart Bw4-I80 (median OS 79.4 months); patients who did receive a surgical treatment or had a HLA-Bw4-T80 gene (2 independent factors) had all a worst OS (median OS 14.4 months, Figure 4). Keeping in mind that CR was found associated with a more activatory NK cell phenotypes and that CR was associated with an increased in OS, our working hypothesis is that inhibitory KIR3DL1/HLA-Bw4-I80 and the absence of KIR2DS5 genotypes found associated with an increased OS were determinants of NK cell subset which has a functional potential for an inhibitory phenotype that is necessary for sustaining disease control and prolonging survival. This observation is similar to those found during acute viral infection; indeed, during, for example, acute human immunodeficiency virus type 1 infection, the NK cell expansion may be first associated with a non specific expansion of activating KIR-expressing NK cells, which however is followed by the occurrence of a protective KIR3DL1+ expressing NK cells, but only in the presence of their putative, HLA-Bw4-I80 ligand [65]. Yet, inhibitory KIR HLA pairs were demonstrated to play a critical role in modulating NK cell function during NK development, indeed the expression and engagement of inhibitory receptors by its ligand is necessary for the transition of CD56^bright^ NK cells to terminally-differentiated and more potent cytotoxic CD56^dim^NK cells [16]; [66] and moreover, inhibitory KIR expression with a prominent role of KIR3DL1/Bw4, necessitate recognizing self HLA during their maturation to fully functional cells with cytotoxic competence and antibody-dependent cell cytotoxicity (ADCC) [66]. Recently, results of KIR/HLA genotyping as outcome predictors of hepatitis C virus-related hepatocellular carcinoma support a central role of cytotoxic NK cells having an inhibitory KIR2DL2/2DL3-HLA-C1+ phenotype in patients showing a favorable outcome [67] as well as in those with spontaneously resolving acute HCV infection [68]. We suppose that in our cases HLA-Bw4-I80 may deliver critical signals to KIR3DL1+ NK cells allowing them to develop into highly functional killers that are able to respond aggressively to target cells, thus resulting in more potent NK cell populations [65]; [69]; [70]. Our data of an increased CR in patients with KIR3DS1 and, then OS, in patients with KIR3DS1 and/or KIR3DS1 plus KIR3DL1 genes that interact with their HLA-Bw4-I80 ligand support this hypothesis (Figure 4).

Moreover, a body of evidence from genetic association studies supports the biological significance not only of the interaction of KIR3DL1 with HLA-Bw4 but also the functional variation seen with different KIR3DL1 and KIR3DS1 and HLA allotypes (reviewed in [71]). In this contest activating (KIR3DS1) and inhibitory (KIR3DL1) receptors may recognize a different set of HLA molecules (HLA-Bw4-I80, HLA-Bw4-T80) and/or different HLA-peptide complex binding [36]; [59]. The dramatic reduction of HLA-T80 molecule, which is known to have a lower affinity for KIR3D receptors, on patient outcome in our series, is showed in the Figures 3C.

Finally, by leading to a reduction in the number of target cells, in accord with previous findings that KIR receptors and HLA-ligands may be more effective when the innate immune response is not likely overwhelmed [57], surgery shows an independent effect of KIR/HLA genotype to OS of patients. KIR3DL1/BW4-I80 genotype leads to increased OS of the patient when a radical surgery had been performed, while the presence of a large mass of tumor cells no treated by surgery flattens the beneficial effect of KIR/HLA genotype to OS.

This is to our knowledge the first study addressing the relevance of KIR genotype on the prognosis of mCRC treated with FOLFIRI regiment. Our results, if confirmed on a larger series of patients and further studies, suggest a central role of NK cells, mainly related to KIR3D/HLA-Bw4 genotype, in the immunoresponse against mCRC. Advantages of this finding are i) the identification of predictive markers of tumor response to chemotherapy from an easily available sample; ii) the relevance of combining KIR/HLA genotype and surgery treatment on the prognosis of mCRC patients rather than surgery alone; iii) the relevance for novel therapeutic strategies for a modulation of the anti-tumor immuno-properties when given with chemotherapeutic agents; iv) to focus on future research of HLA-Bw4/peptide complexes interacting with KIR3D receptors.

Moreover, to more precisely define prognosis and better predict the benefits of adjuvant treatment in mCRC, a quantitative and qualitative assessment of NK cell based on KIR/HLA genotype should be related to known CRC-associated genetic markers (i.e. KRAS mutational status, microsatellite-unstable CRC). Indeed such genetic markers are able to influence the T-cell response in addition to driving cancer growth and progression of CRC [72]; [73]; thus, the combined characterization of both genetic features and NK cell genotypes should identify a group of high-risk patients who would potentially benefit from adjuvant therapy.

## Supporting Information

Table S1 Reports the hazard ratio for the association between characteristic of patients, KIR or/KIR/HLA pairs and overall survival.
(DOC)
