# Correction: Genetic Diversity of the KIR/HLA System and Outcome of Patients with Metastatic Colorectal Cancer Treated with Chemotherapy

**DOI:** 10.1371/journal.pone.0092487

**Published:** 2014-03-10

**Authors:** The PLOS ONE Staff

In the author byline, the name of the sixth author is given incorrectly. The correct name is: Mario D’Andrea
