# Correction: Genetic Diversity of the KIR/HLA System and Outcome of Patients with Metastatic Colorectal Cancer Treated with Chemotherapy

**DOI:** 10.1371/journal.pone.0105850

**Published:** 2014-08-11

**Authors:** The PLOS ONE Staff

There are errors in some of the authors’ affiliations. Please refer to the correct affiliations below:

Dr. Laura Caggiari, Dr. Mariangela De Zorzi and De Re Valli:

Bio-Proteomics Core Facility, Experimental and Clinical Pharmacology, Department of Translational Research (molecular oncology, advanced diagnostics and cell therapies)

Dr Toffoli Giuseppe and Dr. Erika Cecchin:

Experimental and Clinical Pharmacology Unit, Department of Translational Research (molecular oncology, advanced diagnostics and cell therapies).
