# Supplementary material for: Genetic Diversity of the KIR/HLA System and Outcome of Patients with Metastatic Colorectal Cancer Treated with Chemotherapy
Source: PLoS One. 2014 Jan 31;9(1):e84940. doi: 10.1371/journal.pone.0084940 (PMC3908861; doi:10.1371/journal.pone.0084940)
Supplement: Table S1 — Reports the hazard ratio for the association between characteristic of patients, KIR or/KIR/HLA pairs and overall survival. (DOC) [file pone.0084940.s001.doc]

**Table S1. Hazard ratios (HR) for the association between characteristic of patients, KIR or /KIR/HLA pairs and overall survival (OS)**

|  | **HR (95% CI)** |
| --- | --- |
| Sex (male/female) | 1.256 (0.894-1.764) |
| age (<59; 59-65) | 0.853 (0.578-1.260) |
| age (59-65; >65) | 0.874 (0.580-1.318) |
| Tumor location (Rectum/Right colon) | 1.028 (0.683-1.549) |
| Tumor location (Rectum/Left colon) | 1.247 (0.813-1.914) |
| Tumor stage at diagnosis (I-II/III-IV) | 1.367 (0.950-1.966) |
| Radical surgery (yes/no) | 2.065 (1.392-3.064) |
| KIR2DL1 (yes/no) | 0.981 (0.458-2.102) |
| KIR2DL2 (yes/no) | 0.879 (0.630-1.227) |
| KIR2DL3 (yes/no) | 0.849 (0.517-1.393) |
| KIR2DL5 (yes/no) | 1.310 (0.936-1.835) |
| KIR2DL5 (A/B) | 1.235 (0.886-1.723) |
| KIR2DS1 (yes/no) | 1.267 (0.909-1.767) |
| KIR2DS2 (yes/no) | 0.907 (0.650-1.266) |
| KIR2DS3 (yes/no) | 1.121 (0.795-1.580) |
| KIR2DS4 (yes/no) | 0.657 (0.384-1.124) |
| KIR2DS4full (yes/no) | 1.215 (0.842-1.755) |
| KIR2DS4del (yes/no) | 0.693 (0.445-1.079) |
| KIR2DS5 (yes/no) | 1.461 (1.040-2.054) |
| KIR3DS1 (yes/no) | 1.155 (0.827-1.612) |
| R/L KIR2DL1/HLA-C2 | 0.915 (0.389-2.154) |
| R/L KIR2DS1/HLA-C2 | 0.721 (0.504-1.030) |
| R/L KIR2DL2/HLA-C1 | 1.159 (0.829-1.620) |
| R/L KIR2DL3/HLA-C1 | 1.129 (0.796-1.602) |
| R/L KIR2DS2/HLA-C1 | 1.077 (0.770-1.505) |
| R/L KIR3DL1/HLA-Bw4-T80 | 2.792 (1.771-4.402) |
| R/L KIR3DL1-Bw6 | 1.768 (1.172-2.665) |
| R/L KIR3DS1/HLA-Bw4-T80 | 1.568 (0.936-2.628) |
| R/L KIR3DS1/HLA-Bw6 | 0.691 (0.438-1.089) |
| R/L KIR3DL2/HLA-A/03-11 | 1.166 (0.815-1.669) |
| R/L KIR2DS4tot/HLA-C*04 | 1.346 (0.914-1.981) |
| R/L KIR2DS4del/HLA- | 1.391 (0.914-2.120) |
| R/L KIR2DS4del-/HLA- | 1.845 (1.073-3.175) |

**Reference category is each reported as the first name in parenthesis.**

**R/L = presence of both receptor and its cognate ligand**

**^_^ = absence of the HLA molecule**
